# Efficient pH Dependent Drug Delivery to Target Cancer Cells by Gold Nanoparticles Capped with Carboxymethyl Chitosan

**DOI:** 10.3390/ijms15058216

**Published:** 2014-05-09

**Authors:** Alle Madhusudhan, Gangapuram Bhagavanth Reddy, Maragoni Venkatesham, Guttena Veerabhadram, Dudde Anil Kumar, Sumathi Natarajan, Ming-Yeh Yang, Anren Hu, Surya S. Singh

**Affiliations:** 1 Department of Chemistry, University College of Science, Osmania University, Hyderabad, Andhra Pradesh 500007, India; E-Mails:allemadhusudhan@gmail.com (A.M.); bhagavanth.g@gmail.com (G.B.R.); venkateshamchem@gmail.com (M.V.); 2 Department of Laboratory Medicine and Biotechnology, Tzu Chi University, Hualien City 970, Taiwan; E-Mail: ymy74520@gmail.com; 3 Department of Biochemistry, University College of Science, Osmania University, Hyderabad, Andhra Pradesh 500007, India; E-Mails: 74anilkumar@gmail.com (D.A.K.); envy_sumathi@rediffmail.com (S.N.)

**Keywords:** carboxymethyl chitosan, gold nanoparticles, doxorubicin, pH-sensitive drug delivery, cellular uptake, cytotoxicity

## Abstract

Doxorubicin (DOX) was immobilized on gold nanoparticles (AuNPs) capped with carboxymethyl chitosan (CMC) for effective delivery to cancer cells. The carboxylic group of carboxymethyl chitosan interacts with the amino group of the doxorubicin (DOX) forming stable, non-covalent interactions on the surface of AuNPs. The carboxylic group ionizes at acidic pH, thereby releasing the drug effectively at acidic pH suitable to target cancer cells. The DOX loaded gold nanoparticles were effectively absorbed by cervical cancer cells compared to free DOX and their uptake was further increased at acidic conditions induced by nigericin, an ionophore that causes intracellular acidification. These results suggest that DOX loaded AuNPs with pH-triggered drug releasing properties is a novel nanotheraputic approach to overcome drug resistance in cancer.

## 1. Introduction

Cancer is now considered one of the major causes of death worldwide. Amongst various cancer treatments, chemotherapy is a major therapeutic approach. Main obstacles to effective cancer treatment are related to toxicity on healthy proliferating cells and acquisition of multidrug resistance (MDR) [1] against anticancer drugs. Therefore, the selective augmentation of anticancer drug concentrations within tumor tissues remains a major challenge in improving therapeutic efficacy with minimal side effects [2,3]. Although great efforts have been made to overcome MDR, only limited success has been achieved in clinical use [4]. Nanotherapeutics is rapidly progressing and is aimed to solve the problems of conventional chemotherapy. Several nanoparticulate carrier systems including polymeric nanoparticles, dendrimers, liposomes, metal and magnetic nanoparticles are being widely investigated [5,6] especially for drug delivery of siRNA, gene and tumor targeting therapy, bioimaging and biosensing [7–11]. Among these, metallic nanoparticles in general, and gold nanoparticles (AuNPs) in particular, have been gaining eminence in clinical application as unique drug delivery vehicles due to their distinctive shape, size, and surface-dependent properties [12]. In addition to this, their reported biocompatibility [13] and non-cytotoxicity [14] has made drug delivery the leading emerging application of AuNPs [15]. Further, the ease with which their surfaces can be functionalized also makes these nanoparticles attractive for this application [16]. Properly functionalized AuNPs not only can serve as a drug reservoir but also provide long circulation time. Thus nanoparticles have emerged as attractive candidates for delivering various payloads into their targets [17,18]. The payloads could be small drug molecules [19,20] or large biomolecules. This can be amino acids, proteins/enzymes, DNA, RNA [21–25] and other molecular species. The biological activity of the conjugated species such as, their facile bioconjugation and biomodification [26] is not changed in this process. The efficient release of these therapeutic agents at the target site is a prerequisite for effective therapy. Payload release can be triggered by internal (e.g., glutathione (GSH) or pH) or external (e.g., light) stimuli [27,28].

Doxorubicin, a majorly used chemotherapeutic drug, when administered directly, lacks tumor-targeting ability leading to poor distribution and therapeutic effects as well as serious undesirable side effects like cardiotoxicity and myelosuppression [29]. Moreover, as DOX is a hydrophilic molecule, it has restricted transport through the cellular membrane leading to minimal drug internalization. Development of drug resistance is a common limitation with DOX based chemotherapy, especially in ovarian, colon, and breast cancers [30–32]. Chemoresistance is mainly due to over expression of a membrane transporter, p-glycoprotein (pgp) that actively pumps DOX out of the cell [33]. DOX conjugated nanoparticle based drug delivery system evades this mode of efflux as it is taken up by the cells efficiently through endocytosis [34,35]. Colloidal AuNPs incorporating anticancer agents can overcome resistance to drug action, thus reducing the need for higher doses and therefore reducing their ill effects towards normal cells [34,35]. It has been long believed that tumors are acidic in nature through extensive studies using microelectrode measurements [36] or magnetic resonance spectroscopy images [37,38]. Some studies have shown that the extracellular pH of cancerous cells is acidic due to the production of lactic acid but they usually maintain a normal intracellular pH [39]. In general, cancer cells build up acidic environment intracellularly too [40]. In the present study, we made an attempt to synthesize DOX loaded AuNPs that can target cancer cells with intracellular acidic conditions.

Here, we report the synthesis of AuNPs using CM-Chitosan as the reducing and capping agent, and the superior stability of these particles under the conditions of varied pH and electrolyte concentrations is studied. The innovative strategy, presented in this paper is the attachment of the drug molecule through electrostatic or hydrogen bonds that are superior to covalent linkages [41]. Accordingly we report here the synthesis of AuNPs and subsequent DOX loading. The plain AuNPs and DOX loaded AuNPs were characterized in terms of size, zeta potential, Fourier transform infrared spectroscopy (FTIR) method drug-loading efficiency and cytotoxicity. *In vitro* release studies of DOX loaded AuNPs were carried out in solutions of different pH values to demonstrate the effect of pH on the release of DOX from the AuNPs. To evaluate the release of drug in acidic environment we used nigericin to create intracellular acidic environments similar to endosomes and lysosomes in the cells [40]. In addition, methylthialzol tetrazolium (MTT) assay performed on HeLa cells reveals that DOX loaded AuNPs resulted in enhanced cytotoxicity compared to free DOX. Fluorescence based microscopy studies depict an initial localization of DOX in the cytoplasm followed by its subsequent entry in to the nucleus of HeLa cells treated with DOX loaded AuNPs. “Flowcytometry” based studies confirm better release and efficient uptake of DOX from AuNPs under acidic conditions as compared to free DOX. Therefore, DOX loaded AuNPs capped with CM-Chitosan can offer a promising method to enter and release DOX efficiently inside cancer cells, especially in acidic environments.

## 2. Results and Discussion

**Scheme 1.**
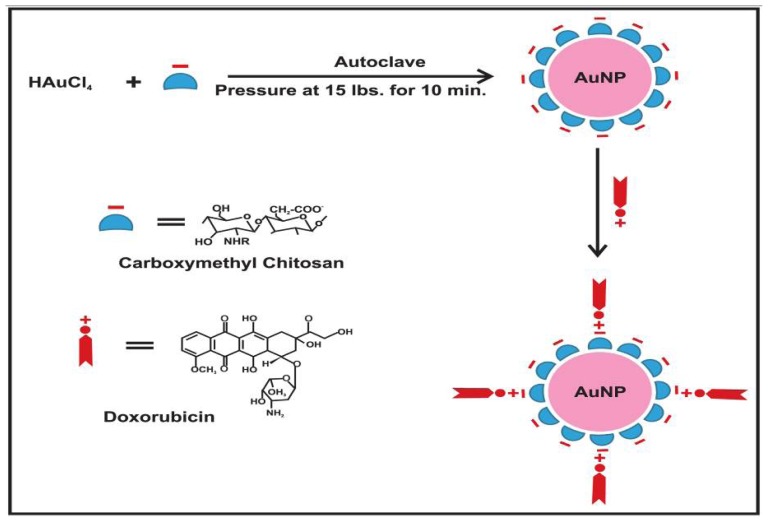
Schematic diagram showing gold nanoparticles (AuNPs) stabilized in CM-Chitosan and subsequent loading of cationic doxorubicin on gold nanoparticles.

CM-Chitosan was used as a reducing as well as a capping agent for the one pot controlled eco-friendly synthesis of AuNPs (Scheme 1). The structure of CM-Chitosan is similar to that of amino acids because it has both amino groups and carboxyl groups in the molecules. The CM-Chitosan is characterized by many carbohydrate units, which make the surface of AuNPs carbohydrate rich. This facilitates the drug loading, especially for drugs like DOX that bear many hydroxyl, amine functional groups capable of forming hydrogen bonds on CM-Chitosan activated AuNPs.

Previous methods utilized for engineering AuNPs, included sodium borohydride or hydrazine hydrate, which are harsh reducing agents. The drawbacks of these methods are lack of sufficient stability in water and formation of aggregates under strong electrolyte conditions and pH changes [42]. To overcome such tedious techniques and to avoid the utilization of harsh reducing agents the interest in this field has shifted towards “green” chemistry and bioprocess approach. These approaches focus on exploration of cost effective eco-friendly and biocompatible reducing agents for synthesis of AuNPs. Early reports cite the use of several plant extracts such as coriander [43], and banana peel [44] for synthesis of AuNPs. Recently, Bacillus subtilis and Rhodopseudomonas capsulate like microorganisms [45] were utilized for the biosynthesis of AuNPs. However, the use of non-toxic and biocompatible naturally occurring polysaccharides such as chitosan [46] and gellan gum [47] for rapid synthesis of AuNPs for drug delivery applications has increased. Various synthesis methods have been employed to fabricate AuNPs by using UV light irradiation [48], and *in situ* process [14]. We tried two ways to get maximum yield of CM-Chitosan capped AuNPs (a) autoclaving at 15 psi pressure for 10 min; and (b) sonication. The autoclaving process gave maximum yield.

### 2.1. Evaluation of Synthesized Gold Nanoparticles

**Figure 1.**
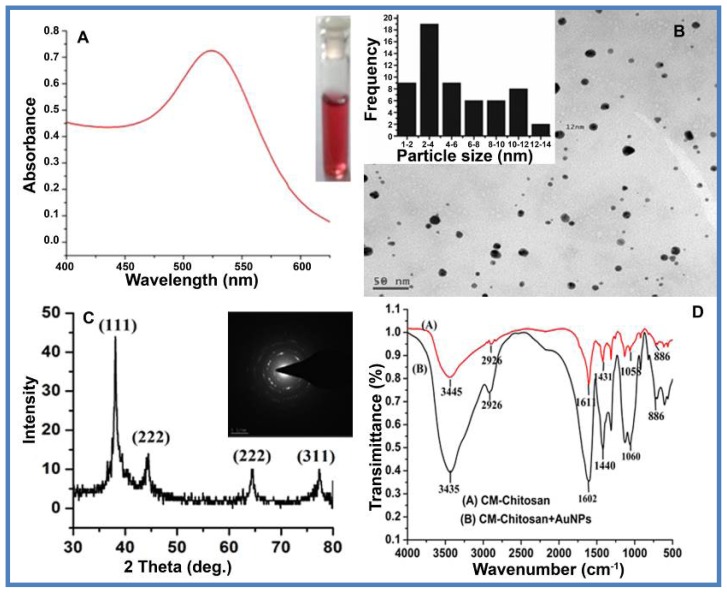
Spectral analysis of AuNPs stabilized in CM-Chitosan (CMC). (**A**) UV-visible spectra of gold nanoparticles (AuNPs) stabilized in CM-Chitosan; (**B**) Transmission electron microscopy (TEM) image of blank gold nanoparticles stabilized by CMC and inset image shows particle size distribution graph; (**C**) X-ray diffraction (XRD) pattern of AuNPs stabilized in CMC, and inset image showing selective electron diffraction pattern of AuNPs stabilized in CMC; and (**D**) Fourier transform infrared spectroscopy (FTIR) spectra of pure CMC and AuNPs stabilized in CMC.

The synthesized AuNPs primarily confirmed by color change from colorless to blushing red were further systematically characterized by UV-visible spectroscopy, powder X-ray diffraction (P-XRD), transmission electron microscopy (TEM), and selected area electron diffraction (SAED) as shown in Figure 1. As predicted, AuNPs exhibit SPR (surface plasmon resonance) at 520 nm (Figure 1A). This peak is indicative of the presence of anisotropic AuNPs. The peak position of AuNPs did not change, indicating there is no agglomeration of the nanoparticles [49]. The UV-visible spectra of AuNPs prepared by varying the concentrations of HAuCl_4_ (0.1 to 1 mM), CM-Chitosan (0.1 to 1 mM) and varying the time (2–10 min) are shown in Figure S1. The efficiency of nanoparticle synthesis increases with increase in the concentration of HAuCl_4_, CM-Chitosan and autoclave time due to an enhancement in the oxidation of hydroxyl groups of CM-Chitosan by gold ions. TEM images (Figure 1B) showed that the CM-Chitosan stabilized AuNPs were spherical in shape and are well distributed in the polymer matrix. Histogram (Insert, Figure 1B) shows the particle size distribution of gold nanoparticles to be 9 ± 2 nm. The size of AuNPs obtained corresponds to the ideal size necessary for drug delivery applications. It has been reported that optimum efficiency of delivery into the pulmonary system can be achieved with nanoparticles of diameter <50 nm [50]. Enhanced uptake efficiency has also been shown for gastrointestinal absorption [51] with particle sizes of around 50 to 100 nm. The XRD technique was used to determine the crystal structure of eco-friendly synthesized AuNPs. Figure 1C displays the XRD pattern of the synthesized AuNPs. A number of Bragg reflections with 2θ values of 38.25, 43.95, 64.5 and 77.21 corresponding to the (111), (200), (220) and (311) sets of lattice planes respectively, therefore indexed as the band for face centered cubic (fcc) structure of AuNPs. The peak corresponding to the (111) plane is most intense compared to the other planes. The broadening of these peaks is mostly due to the effect of nano-sized particles. Insert of Figure 1C shows the SAED pattern of AuNPs, exhibiting polycrystalline diffraction rings, which can be indexed to cubic-phase metal gold, indicating that these nanoparticles are crystalline metallic gold. The evidence for the reduction of HAuCl_4_ to AuNPs by using CM-Chitosan was obtained from the FTIR spectra. The FTIR spectra of pure CM-Chitosan and AuNPs/CM-Chitosan were recorded and represented in Figure 1D. The major peaks can be allotted as follows: 3435 cm^−1^ (–OH and N–H), 2926 cm^−1^ (C–H), 1611 cm^−1^ (the coupling of C=0 and N–H), 1431 cm^−1^ (the coupling of C–N and N–H), 1058 cm^−1^ (C–O) and 886 cm^−1^ (ring). As presented in Figure 1D, the spectrum of the CM-Chitosan reduced gold nanoparticles in comparison with that of CM-Chitosan shows an increase in the intensity and blue-shift of the C=O and N–H bend vibrations (1602 cm^−1^). There are two possible reasons for the change in the spectrum. One reason might be the electrostatic interaction between charged AuNPs and –COO^−^ groups of CM-Chitosan. Another reason could be the formation of a coordination bond between AuNPs and nitrogen/oxygen atom in CM-Chitosan molecules.

**Figure 2.**
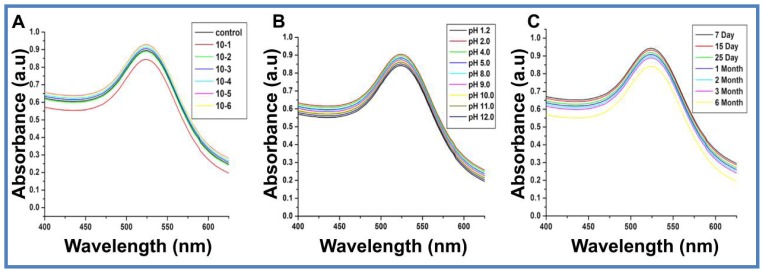
UV-visible spectra of AuNPs depicting their high stability due to the influence of CMC capping. Stability of CMC stabilized AuNPs were tested against (**A**) different electrolytic (NaCl) concentration; (**B**) Varied pH conditions; and (**C**) Six month stability study showing no aggregation. The data is representative of three independently conducted experiments.

Stability of nanoparticles in solution is an important requirement for their therapeutic and biomedical application. This feature was analyzed by monitoring the SPR under different pH and electrolytic conditions over a reasonable period of time. The bathochromic shift normally observed in UV-visible spectra is an indication of an increase in the size of the particle or agglomeration of nanoparticles or both. The synthesized nanoparticles did not show any significant change in the intensity or position of the absorbance at 520 nm with increase in the concentration of electrolyte to 1 × 10^−2^ M (NaCl) (Figure 2A). pH range of 1.2 to 12 did not alter the size of NPs and no major agglomeration was observed (Figure 2B). The CM-Chitosan stabilized AuNPs were stable up to six months at room temperature (Figure 2C) due to the capping of CM-Chitosan. The above observations indicate their stability for drug-delivery applications.

### 2.2. Evaluation of DOX Loaded Gold Nanoparticles

**Figure 3.**
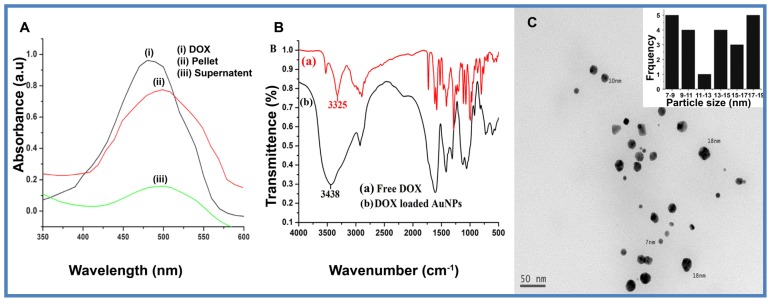
Spectral analysis of DOX loaded AuNPs. (**A**) UV-visible spectra of native DOX solution, DOX bound to AuNPs and unbound DOX; (**B**) FTIR spectra of (a) native DOX and (b) DOX loaded AuNPs; and (**C**) TEM image of DOX loaded AuNPs and inset image showing particle size distribution graph.

DOX loaded AuNPs were next analyzed by UV-visible absorbance to deduce the amount of DOX loading. DOX was added to the 2 mL of AuNPs as described in the methods section. The percentage loading efficiency of DOX on AuNPs was determined based on DOX content in the pellet obtained and it was found to be 83.3% ± 4% of DOX (83 μg in 2 mL) (Figure 3A). At neutral pH, the zeta potential of CM-Chitosan reduced AuNPs was determined to be −21.6 mV due to the AuNPs being covered with the anionic CM-Chitosan, during synthesis. The zeta potential of DOX (p*K*_a_ = 8.2) loaded AuNPs was −10.60 mV and the results shown in Figure S2A,B. The decrease in the zeta potential is attributed to the presence of positively charged DOX. The small decrease in the charge even at 83.3% loading of DOX indicates that other attractive forces including hydrogen bonding could be playing a major role in facilitating the drug-loading process. The hydrogen bonding between protonated amine groups of the DOX molecule with CM-Chitosan on the surface of AuNPs is evident by FTIR, where the NH stretching band at 3325 cm^−1^ of free DOX shifted to 3438 cm^−1^ in case of DOX loaded AuNPs (Figure 3B). The TEM images of DOX loaded AuNPs indicate significant change in particle size (Figure 3C).

### 2.3. Efficient DOX Release from Loaded AuNPs at Acidic pH

**Figure 4.**
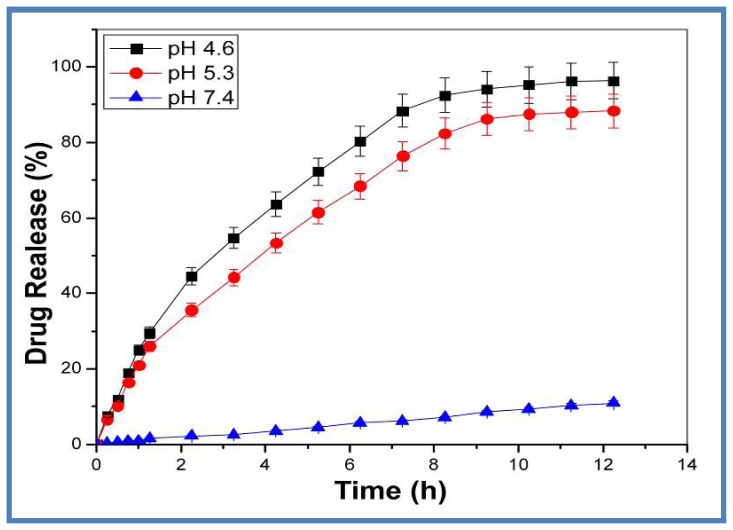
pH dependendent release of DOX from DOX loaded nanoparticles. DOX release studies were carried out by calculating the amount of DOX in acetate (pH 4.6 and 5.3) and phosphate (pH 7.4) buffer dialysis filtrate at different time intervals. The data expressed is mean ± S.D. where *n* = 3.

DOX loaded AuNPs were exposed to physiological (pH 7.4) and acidic (acetate buffer pH 4.6 and 5.3) conditions at 37 °C to measure the release of DOX *in vitro* and evaluate the feasibility of using DOX loaded AuNPs to target cancer tissue. As shown in Figure 4, the rate and amount of DOX released from the DOX loaded AuNPs were strongly dependent on the pH of the medium. DOX loaded AuNPs showed much faster DOX release at pH 4.6 and 5.3 than at pH 7.4. At the end of 12.25 h, 96.6% ± 3.2%, 88.82% ± 2.3% and 10.46% ± 2.5% of DOX was released in acetate and phosphate buffer respectively. The pH dependent release may help to improve efficiency of DOX loaded nanoparticles as efficient delivery system, where normal cells are not affected. Based on previous studies, it is believed that DOX is internalized by cells through the endocytosis process [52]. The acidic environment in the endosome may trigger the rapid release of DOX from the DOX loaded AuNPs, thereby greatly enhancing the cell cytotoxicity. Previous studies have reported the release of DOX from carbon nanotubes, occurring inside cell endosomes and lysosomes under acidic conditions [53,54]. Also, negligible release of DOX from DOX loaded AuNPs under physiological pH will help to reduce toxicity of DOX to the normal tissue since the pH of body fluids is maintained around pH 7.4 [55,56]. It is expected that the DOX-loaded AuNPs will accumulate in the tumor tissue preferentially through the EPR (Enhanced Permeability and Retention) effect. Once in the tumor tissue, these DOX loaded AuNPs will be internalized by the tumor cells, largely via folate-receptor-mediated endocytosis, and will be located in the acidic endosomal compartments where DOX could be cleaved from the AuNPs and subsequently diffuse into the cytosol and later into the nucleus [57]. The endocytic compartments of the tumor cells can help to quickly provide a sufficient level of DOX in the tumor tissue/cells, thereby greatly enhancing the efficacy of anti-cancer drugs.

### 2.4. Dynamic Light Scattering Size Data

Dynamic light scattering (DLS) is the most useful techniques for measuring the sizes, and size distributions, of nanoparticles in liquids. DLS measurements were performed with CM-Chitosan capped AuNPs suspension before and after addiction of DOX. The hydrodynamic diameter of CM-Chitosan capped AuNPs was equal to 33.09 ± 1 nm (polydispersity index, PDI 0.46) as shown in Figure S3A. After addition of DOX (Figure S3B) the hydrodynamic diameter of DOX loaded AuNPs 65.57 ± 4 nm (polydispersity index, PDI 0.459) gets much greater than that of individual of CM-Chitosan capped AuNPs. This result clearly indicates the loading of DOX on AuNPs.

### 2.5. In Vitro Cytotoxicity Is Enhanced with DOX Loaded AuNPs

**Figure 5.**
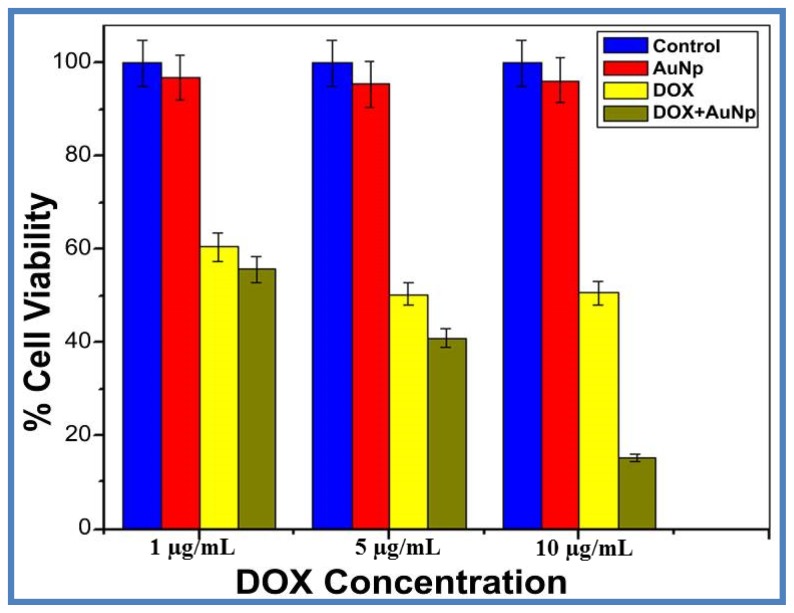
Cytotoxicity of free and AuNPs loaded DOX in Hela cells. HeLa cells (5 × 10^3^ cells) were seeded in 96 well plates and after 70% confluency treated with different concentrations of DOX or DOX loaded AuNPs in phosphate buffered saline (PBS) and grown for further 24 h. Cell viability was determined by MTT assay. Data is expressed is mean ± S.D. where *n* = 3.

We further evaluated the cytotoxicity of DOX loaded AuNPs in HeLa cells. Untreated cells and free AuNPs treated cells show up to 95% ± 5% cell viability at 1, 5 and 10 μg/mL concentration of AuNPs. Thus the cytotoxic effect of free AuNPs is negligible. Figure 5 shows the viability of HeLa cells after exposure to free DOX and DOX loaded AuNPs for 24 h. At the end of 24 h, DOX loaded AuNPs demonstrated enhanced cytotoxicity, with just 11% ± 2% survival at 10 μg/mL compared to free DOX treated cells where nearly 50% ± 0.5% survival was noted at the same concentration. The increased cytotoxicity of DOX loaded AuNPs may be due to efficient transport of DOX by nanoparticles through an endocytosis mechanism compared to the passive diffusion mechanism of free DOX in to cells [52]. AuNPs by themselves showed no significant toxicity on cells.

### 2.6. Cells Released DOX from DOX Loaded AuNPs More Effectively Than Free DOX under Intracellular Acidic Conditions

**Figure 6.**
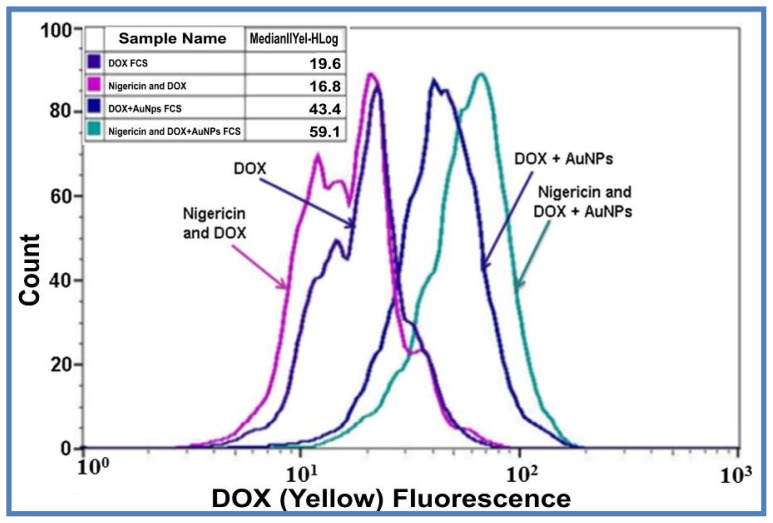
Nigericin induced acidification and effect on uptake of free DOX and DOX loaded AuNPs uptake by Hela cells *in vivo*. Cellular uptake of DOX and DOX loaded AuNPs by HeLa cells after intracellular acidic environment created by 2.5 μM Nigericin, as measured by Millipore Guava 8HT easycyte flowcytometer after 30 min incubation. Inset image shows DOX Median Fluorescence Intensity (MFI) values. The data is representative of two independent experiments.

Further, it is well documented that cancer cells have an extracellular acidic environment [58] and free DOX is not effectively taken up at acidic condition [41]. To investigate the effect of pH on loaded DOX uptake and release in cells, we treated cells with an ionophore, nigericin, which causes intracellular acidification by allowing entry of protons in exchange for K^+^ ions. “Flowcytometry” analysis for DOX fluorescence indicated an increased DOX release from DOX loaded AuNPs when HeLa cells were pretreated for 30 min with 2.5 μM nigericin, before the addition of the drug. The data presented in Figure 6 indicates intensity of DOX fluorescence with and without pretreatment with nigericin. Native DOX uptake decreased from 19.6 to 16.8 AU. This data suggests that free DOX uptake by cancer cells in reduced with nigericin treatment and corroborates with earlier studies that in acidic environments DOX uptake is inhibited [57]. DOX loaded AuNPs are a better vehicle for DOX uptake where fluorescence increased from 19.6 to 43.4 AU, a 2.2-fold increase in drug uptake in normal physiological conditions. DOX uptake increased from 16.8 (free DOX) to 59.1 AU for DOX loaded AuNPs, corresponding to a significant 3.5-fold increase in DOX uptake under intracellular acidic conditions. DOX being a weakly basic drug is better absorbed with high extracellular and intracellular pH in HCT116 colon cancer cells [59]. We also observed that DOX alone could not effectively enter HeLa cervical cancer cells with low intracellular pH. Since many cancer cells have low intracellular pH [40], drugs should be able to work at the pH within the cells. Here, we show that DOX loaded AuNPs work effectively in intracellular acidic environment and induce cell death much more efficiently. The observations indicate that acidic environments inside the cancer cells make loaded DOX more effective in its pharmacological action as compared to free DOX.

**Figure 7.**
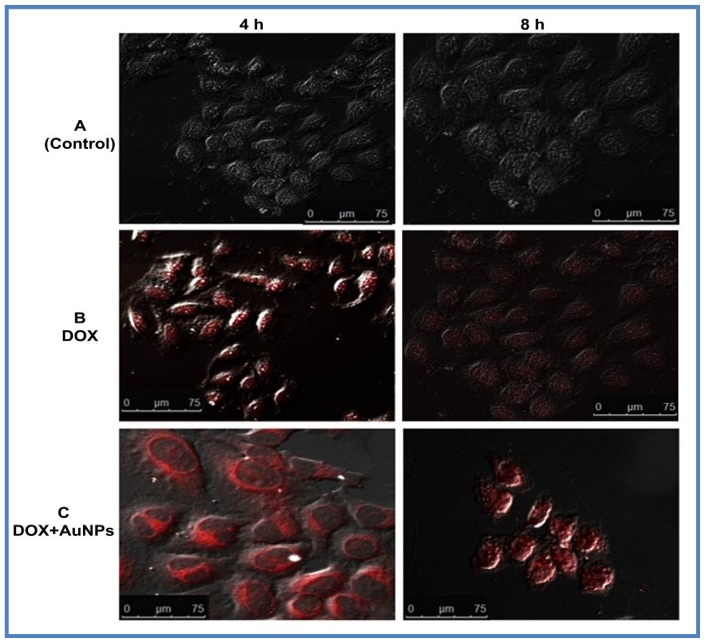
Confocal microscopy images depicting intracellular/nuclear localization of DOX in HeLa cells. HeLa cells were treated with 10 μg/mL DOX and DOX loaded AuNPs for 4 and 8 h followed by confocal imaging with Leica TCS SP5-II microscope at 200× magnification to interpret the cellular localization of DOX, scale 75 μm. (**A**) Control HeLa cells; (**B**) HeLa cells treated with DOX; and (**C**) and DOX loaded AuNPs. Data is representative of three independent experiments.

Further internalization and cellular localization of DOX and DOX loaded AuNPs in HeLa cells were assessed by intrinsic fluorescence of DOX with confocal microscopy. The DOX loaded AuNPs are internalized by the cells at a time similar to that of DOX. However, as compared to native DOX, which directly enters the nucleus after cellular uptake, DOX loaded AuNPs showed an initial localization in the cell cytoplasm followed by the release of DOX and its entry into the nucleus (Figure 7). The DOX loaded AuNPs accumulated at the perinuclear region at 4 h treatment when compared to native DOX that showed overall cellular distribution at the same time point. At 8 h, DOX was seen localized in the nucleus after its release from AuNPs and the cells showed deformed status inferring that DOX was entering the cell at a faster rate when compared to native DOX. The apoptosis induced by DOX loaded AuNPs was clearly visible as the cells shrank to a spherical shape [41]. This was in accordance with previous studies, which used thiolated methoxy polyethylene glycol (MPEG-SH) stabilized AuNPs as delivery vehicle for DOX [55].

The objective behind devising biogenic DOX loaded AuNPs based drug delivery system is the sustained release of DOX in an ideal tumor microenvironment. This was clearly shown in drug uptake studies where the uptake of loaded DOX increased with time allowing for sustained release of the drug for a period of 8 h, unlike DOX, which showed a similar uptake for 4 and 8 h. The sustained pH based release was verified by creating an ideal acidic environment, using an ionophore nigericin, which resulted in a 3.5-fold increase when compared to DOX alone. The *in vitro* drug release studies showed 96% and 88% drug release from loaded DOX in acetate buffer (pH 4.6 and 5.3 respectively) and only 10% drug release at physiological pH of 7.4. This type of pH control and targeted drug release mechanism could be an excellent tool for improvised cancer therapy. The DOX loaded onto AuNPs by a low pH labile CMC linkage is severed upon acidification in the endosomes following its uptake by endocytosis. DOX upon its release from the drug delivery vehicle enters the cell nucleus in a time dependent manner (Figure 7), evading the p-glycoprotein mode of drug efflux. Thus, this drug delivery system is excellent for use in MDR cells overexpressing p-glycoprotein, as it evades this resistance mechanism by slow nucleus targeted drug release. The low pH based release of the drug from the nanoparticle conjugated system within the endosomes coupled with the enhanced permeability and retention effect (EPR) [60,61] of cancerous tissues ensures selective toxicity only to these cells. It will be worthwhile to study the effect of DOX loaded nanoparticles under extracellular acidic conditions and in animal models for chemotherapy. Solid tumors exhibiting low stromal pH [58] might cause DOX to be released from the loaded AuNPs outside the cells, subjecting the cells to free DOX. However, from our flow cytometry data it is evident that cellular uptake of DOX loaded AuNPs start as early as 30 min to 1 h of treatment (Figure 6). Also the drug release up to acidic pH (4.6 and 5.3) was only 18% ± 2% up to 2 h and increased to 27% ± 3% by 3 h (Figure 4), indicating that majority of the DOX loaded AuNPs are still available to permeate through the cells and function accordingly.

## 3. Experimental Section

### 3.1. Materials

Doxorubicin hydrochloride was a gift from Natco Pharma Ltd. (Hyderabad, India). Hydrochloroauric acid (HAuCl_4_) was purchased from Sigma-Aldrich (St. Louis, MO, USA). CM-Chitosan was prepared according to the procedure given in the literature [62]. Briefly, Chitosan (10 g), sodium hydroxide (12.5 g) and isopropanol solvent (100 mL) were suspended in a flask to swell and alkalize at room temperature for 1 h. The temperature was maintained at 25 °C in a water bath. The monochloroaceticacid (13 g) was dissolved in isopropanol (50 mL), and added to the reaction mixture drop wise within 30 min and the reaction was allowed to take place for 4.5 h at 55 °C. Then the reaction was stopped by adding a few drops of acetic acid to neutralize the reaction mass and the isopropanol were decanted. Ethyl alcohol (80%) was added and the solid product was filtered and rinsed with 80% ethyl alcohol to desalt and dewater. The product was then vacuum-dried at 40 °C.

Human cervical carcinoma cell line (HeLa) was obtained from National Center for Cell Science (NCCS) (Pune, India). Dulbecco’s PBS (pH 7.4) and MTT were purchased from HiMedia Laboratories (Mumbai, India); DMSO (Spectroscopic grade) and CC mount were purchased from Sigma-Aldrich (Bangalore, India) and paraformaldehyde from Merck India Ltd., (Mumbai, India). All other chemicals were of analytical grade and were obtained from Merck India Ltd., (Mumbai, India).

### 3.2. Cell Culture

HeLa (Human cervical cancer) cells were maintained in RPMI-1640 medium supplemented with 4.5 g/L d-glucose, 1 mM sodium pyruvate, 1.5 g/L sodium bicarbonate and 10% fetal calf serum (HiMedia Lab, Mumbai, India). The cells were maintained at humidified atmosphere, 37 °C and 5% CO_2_ in an incubator (Mumbai, India).

### 3.3. Synthesis of AuNPs

Glassware was cleaned in a bath of freshly prepared aquaregia solution (HCl:HNO_3_ 3:1) and then rinsed thoroughly with H_2_O prior to use. Before the preparation of AuNPs, the stock solution of 1% (1 g) CM-Chitosan was prepared in Millipore water. The solution was stirred overnight and turned into a homogeneous system. An aqueous solution of HAuCl_4_ (1 mL, 1 mM) was mixed with a diluted solution of CM-Chitosan (3 mL, varied concentration) and added in a boiling tube. The boiling tube was sealed with aluminum foil and kept in an autoclave. AuNPs are prepared by varying the time of autoclaving at 15 psi pressure and 120 °C temperature. AuNPs are obtained by autoclaving for 10 min at 15 psi pressure and 120 °C temperature varying the concentrations of HAuCL_4_ and CM-Chitosan. The colorless reaction mixture was converted to the characteristic clear blushing red color after autoclaving. The appearance of color was indicated the formation of AuNPs.

### 3.4. Loading of DOX onto Gold

A calculated amount of DOX was added to AuNPs dispersion, obtained as described above, resulting in a final DOX concentration of 10^−4^ M (100 μg in 2 mL). The DOX solution was added to AuNPs dispersion and stirred at 1000 rpm for 30 min. The mixture of DOX and AuNPs dispersion was incubated for 24 h at room temperature for complete loading of drug on nanoparticles and then centrifuged at 20,000 rpm for 30 min. The obtained pellet after centrifugation was separated from the supernatant solution and redispersed in deionized water prior to further characterization. The DOX concentration in redispersed pellet was determined by measurements of its UV absorbance at 480 nm using UV-visible spectroscopy and the percentage loading of DOX on AuNPs was estimated by following formula:

$$\%\;\text{Loading}=\frac{\text{Total}\;\text{amount of DOX added}-\text{Amount of DOX}\;\text{in redispersed pellet}}{\text{Total amount of }\text{DOX added}}\times 100\%$$

### 3.5. Characterization of Materials

The change in surface plasmon resonance (SPR) of CM-Chitosan capped AuNPs, before and after loading of DOX, was monitored by UV-visible spectroscopy measurements carried out on a Dual Beam UV-visible Spectrophotometer (Shimadzu-3600, Kyoto, Japan). X-ray diffraction (XRD) measurement of CM-Chitosan stabilized AuNPs was carried out by preparing films of nanoparticle dispersion on glass substrates by simple solvent evaporation method at room temperature. The diffraction measurements were carried out on X’pert Pro X-ray diffractometer (PANalytical B.V., Eindhoven, The Netherlands) instrument operating at 40 kV and a current of 30 mA at a scan rate of 0.388/min^−1^.

The morphology and size distribution of the CM-Chitosan capped AuNPs and DOX loaded AuNPs dispersion was carried out by transmission electron microscopy (TEM) measurement; casting nanoparticle dispersion on carbon-coated copper grids and allowed to dry at room temperature. Measurements were done on TECHNAI G2 F30 S-TWIN instrument (FEI Company, Hillsboro, OR, USA) operated at an accelerated voltage of 200 kV with a lattice resolution of 0.14 nm and point image resolution of 0.20 nm. To obtain size distributions of AuNPs, approximately 60 particles were counted and then converted into histograms. The surface charge and zeta potential of AuNPs before and after loading of DOX was determined by using the Zeta sizer 300 HAS (Malvern, Worcestershire, UK) as such without dilution. Fourier transform infrared (FTIR) spectra of native CM-Chitosan, CM-Chitosan capped AuNPs, native DOX and DOX loaded AuNPs were recorded in KBr pellets using FTIR spectrophotometer (Bruker optics**-**TENSOR 27, Karlsruhe, Germany). The scan was performed in the range of 400–4000 cm^−1^.

### 3.6. In Vitro Drug Release Study of DOX Loaded Gold Nanoparticles

The release studies were carried out in a glass apparatus at 37 °C in acetate buffer (pH 4.6 and 5.3) and phosphate buffer (pH 7.4) solutions. First the dialysis tube containing 2 mL of DOX loaded AuNPs (DOX concentration 552 μg in 2 mL) was shifted to a beaker containing 100 mL of phosphate buffer (pH 7.4) with continuous stirring at 100 rpm. Sink condition was maintained by periodically removing 5 mL samples and substituting equal volume of buffer. The amount of DOX released was analyzed with a spectrophotometer at 480 nm. A similar release study was carried out in acetate buffer (pH 4.6 and 5.3). The drug release studies were performed in triplicate for each of the samples. Standard deviation was calculated using origin pro 7.5 plots.

### 3.7. Drug Uptake Studies

HeLa cells (1 × 10^6^ cells) were taken in 5 mL of RPMI medium in sterile wide bottom centrifuge tubes after counting on Bright Line Haemocytometer (Sigma Ltd., Buffalo, NY, USA). The cells were serum starved for 16 h. Then the medium was replaced with 5 mL fresh plain medium containing native DOX and DOX loaded AuNPs (prepared in PBS) at concentration of 10 μg/mL. Ionophore based drug uptake assay was carried out in a similar manner by pretreating cells with 2.5 μM nigericin for 30 min and further incubating for another 30 min with 10 μg/mL of DOX and DOX loaded AuNPs. The data was acquired with flowcytometer (Millipore Guava 8HT easycyte, Hayward, CA, USA). 10,000 events were acquired with flow rate 0.59 μL/s. The fluorescence quantitation was done (using yellow filter) with the express pro software. The data analysis was performed by FlowJo software (version 7.6, Tree Star Inc., Ashland, OR, USA).

### 3.8. Confocal Microscopy

For confocal microscopy, HeLa cells were grown on cover glass (22 × 30 mm rectangular No.1 Corning^®^ (Corning, Berlin, Germany) cover glasses) in petriplates (Nunc) until semiconfluent growth was achieved. After 16 h starvation, cells were replaced with fresh plain medium containing native DOX and DOX loaded AuNPs (prepared in PBS) at a concentration of 10 μg/mL and incubated for 4 and 8 h along with a set of untreated cells. At different time intervals, the cells were washed in PBS and fixed using 4% paraformaldehyde for 15 min at room temperature. The cover slips were mounted onto slides using CC mount (Sigma Ltd., St. Louis, MO, USA). The cells were then imaged with a confocal microscope (Leica TCS SP5-II, Heidelberg, Germany) at an emission wavelength of 590–610 nm (excitation laser 543 nm) with 200× magnification.

### 3.9. Cytotoxicity Studies

HeLa cells were seeded on flat bottom 96 well plates (Orange Scientific, Braine-l’Alleud, Belgium) at 5 × 10^3^ cells/well and cultured at 37 °C and 5% CO_2_. After 24 h, the cells were serum starved overnight. The CM-Chitosan capped AuNPs, native DOX and DOX loaded AuNPs (prepared in PBS) were added at a concentration range of 1–10 μg/mL in a total volume of 200 μL. After addition, the plate was further incubated at 37 °C and 5% CO_2_ for 24 h. The cytotoxicity was then tested by the addition of MTT (3-(4,5-dimethylthiazolyl-2)-2,5-diphenyltetrazolium bromide) prepared in culture medium at a working concentration of 0.4 mg/mL. The plate was further incubated for 2 h so that the MTT is reduced by the live cells to produce a purple formazan product. After this time, the medium was aspirated and 200 μL of DMSO was added to each well. The plate was agitated gently for 5 min before measuring the optical density at 570 nm in each well using MultiSkan EX Elisa multiplate reader (Thermo Scientific, Shanghai, China). The experiment was performed in triplicate. Data was statistically analyzed using was calculated using origin pro 7.5 plots.

### 3.10. Statistical Analysis

Statistical analysis was performed using origin pro 7.5 plots wherever applicable. Flow cytometry data was analyzed by using FlowJo software.

## 4. Conclusions

With ever increasing necessity to develop drugs with greater efficiency to target cancer cells specifically and improve chemosensitivity, there is a constant requirement to develop or improve drug delivery strategies. Therefore, we have tried in this study to develop a novel nanoparticle synthesis procedure that facilitates drug loading and preferentially makes cancer cells more responsive. In this study, we demonstrate size controlled eco-friendly one pot synthesis of AuNPs by using CM-Chitosan as a capping and reducing agent to be a resourceful and an inexpensive method therefore providing an advantage over other traditional methods of synthesis of conjugated chemotherapeutic agents. The gold nanoparticles capped with CM-Chitosan exhibit stability in a wide range of pH and electrolyte concentration. Further, these nanoparticles loaded with the cytotoxic drug DOX are highly stable without leaching. Efficient uptake of DOX loaded gold nanoparticles at physiological pH and response at intracellular acidic pH make them an ideal drug delivery system to target cancers.

## Supplementary Information



## References

[1] Gottesman M.M., Fojo T., Bates S.E. (2002). Multidrug resistance in cancer: Role of ATP-dependent transporters. Nat. Rev. Cancer.

[2] Maeda H. (2001). SMANCS polymer-conjugated macromolecular drugs: Advantages in cancer chemotherapy. Adv. Drug Deliv. Rev.

[3] Oishi M., Hayashi H., Iijima M., Nagasaki Y. (2007). Endosomal release and intracellular delivery of anticancer drugs using pH-sensitive PEGylated nanogels. J. Mater. Chem.

[4] Szakacs G., Paterson J.K., Ludwig J.A., Genthe C.B., Gottesman M.M. (2006). Targeting multidrug resistance in cancer. Nat. Rev. Drug Discov.

[5] Vlerken L.E.V., Amiji M.M. (2006). Multi-functional polymeric nanoparticles for tumour-targeted drug delivery. Expert Opin. Drug Deliv.

[6] Han G., Ghosh P., Rotello V.M. (2007). Functionalized gold nanoparticles for drug delivery. Nanomedicine.

[7] Qi L., Wu L., Zheng S., Wang Y., Fu H., Cui D.X. (2012). Cell-penetrating magnetic nanoparticles for highly efficient delivery and intracellular imaging of siRNA. Biomacromolecules.

[8] Pan B.F., Cui D.X., Sheng Y., Ozkan C.G., Gao F., He R., Li Q., Xu P., Huang T. (2007). Dendrimer-modified magnetic nanoparticles enhance efficiency of gene delivery system. Cancer Res.

[9] Huang P., Li Z.M., Lin J., Yang D.P., Gao G., Xu C., Bao L., Zhang C.L., Wang K., Song H. (2011). Photosensitizer-conjugated magnetic nanoparticles for *in vivo* simultaneous magnetofluorescent imaging and targeting therapy. Biomaterials.

[10] Nowicka A.M., Kowalczyk A., Jarzebinska A., Donten M., Krysinski P., Stojek Z. (2013). Progress in targeting tumor cells by using drug magnetic nanoparticle conjugated. Biomacromolecules.

[11] Cheng M.M.C., Cuda G., Bunimovich Y.L., Gaspari M., Heath J.R., Hill H.D., Mirkin C.A., Nijdam A.J., Terracciano R., Thundat T. (2006). Nanotechnologies for biomolecular detection and medical diagnostics. Curr. Opin. Chem. Biol.

[12] Ghosh P., Han G., De M., Kim C.K., Rotello V.M. (2008). Gold nanoparticles in delivery applications. Adv. Drug Deliv. Rev.

[13] De M., Ghosh P.S., Rotello V.M. (2008). Applications of nanoparticles in biology. Adv. Mater.

[14] Huang L., Zhai M., Peng J., Xu L., Li J., Wei G. (2007). Synthesis, size control and fluorescence studies of gold nanoparticles in carboxymethylated chitosan aqueous solutions. J. Colloid Interface Sci.

[15] Kattumuri V., Katti K., Bhaskaran S., Boote E.J., Casteel S.W., Fent G.M., Robertson D.J., Chandrasekhar M., Kannan R., Katti K.V. (2007). Gum arabic as a phytochemical construct for the stabilization of gold nanoparticles: *In vivo* pharmacokinetics and X-ray-contrast-imaging studies. Small.

[16] Shan J., Tenhu H. (2007). Recent advances in polymer protected gold nanoparticles: Synthesis, properties and applications. Chem. Commun.

[17] Paciotti G.F., Myer L., Weinreich D., Goia D., Pavel N., McLaughlin R.E., Tamarkin L. (2004). Colloidal gold: A novel nanoparticle vector for tumor directed drug delivery. Drug Deliv.

[18] Hong R., Fischer N.O., Verma A., Goodman C.M., Emrick T., Rotello V.M. (2004). Control of protein structure and function through surface recognition by tailored nanoparticle scaffolds. J. Am. Chem. Soc.

[19] Kim C.K., Ghosh P., Pagliuca C., Zhu Z.J., Menichetti S., Rotello V.M. (2009). Entrapment of hydrophobic drugs in nanoparticle monolayers with efficient release into cancer cells. J. Am. Chem. Soc.

[20] Selvakannan P., Mandal S., Phadtare S., Gole A., Pasricha R., Adyanthaya S.D., Sastry M. (2004). Water-dispersible tryptophan-protected gold nanoparticles prepared by the spontaneous reduction of aqueous chloroaurate ions by the amino acid. J. Colloid Interface Sci.

[21] Chithrani B.D., Chan W.C.W. (2007). Elucidating the mechanism of cellular uptake and removal of protein-coated gold nanoparticles of different sizes and shapes. Nano Lett.

[22] Visaria R.K., Griffin R.J., Williams B.W., Ebbini E.S., Paciotti G.F., Song C.W., Bischof J.C. (2006). Enhancement of tumor thermal therapy using gold nanoparticle-assisted tumor necrosis factor-alpha delivery. Mol. Cancer Ther.

[23] Cheng Y., Samia A.C., Meyers J.D., Panagopoulos I., Fei B.W., Burda C. (2008). Highly efficient drug delivery with gold nanoparticle vectors for *in vivo* photodynamic therapy of cancer. J. Am. Chem. Soc.

[24] Ghosh P.S., Kim C.K., Han G., Forbes N.S., Rotello V.M. (2008). Efficient gene delivery vectors by tuning the surface charge density of amino acid-functionalized gold nanoparticles. ACS Nano.

[25] Lee J.S., Green J.J., Love K.T., Sunshine J., Langer R., Anderson D.J. (2009). Gold, poly(β-amino ester) nanoparticles for small interfering RNA delivery. Nano Lett.

[26] Katz E., Willner I. (2004). Integrierte hybrid systeme aus nanopartikeln und biomolekülen: Synthese, eigenschaften and anwendungen. Angew. Chem.

[27] Hong R., Han G., Fernandez J.M., Kim B.J., Forbes N.S., Rotello V.M. (2006). Glutathione-mediated delivery and release using monolayer protected nanoparticle carriers. J. Am. Chem. Soc.

[28] Polizzi M.A., Stasko N.A., Schoenfisch M.H. (2007). Water-soluble nitric oxide-releasing gold nanoparticles. Langmuir.

[29] Kenneth F.H., Annemette V.T., Maxwell S., Peter B.J. (2005). Dexrazoxane protects against myelosuppression from the DNA cleavage-enhancing drugs etoposide and daunorubicin but not doxorubicin. Clin. Cancer Res.

[30] Schondorf T., Kurbacher C.M., Gohring U.J., Benz C., Becker M., Sartorius J., Kolhagen H., Mallman P., Neumann R. (2002). Induction of *MDR1*-gene expression by antineoplastic agents in ovarian cancer cell lines. Anticancer Res.

[31] Linn S.C., Giaccone G. (1995). MDR1/P-glycoprotein expression in colorectal cancer. Eur. J. Cancer.

[32] Yu S.T., Chen T.M., Tseng S.Y., Chen Y.H. (2007). Tryptanthrin inhibits MDR1 and reverses doxorubicin resistance in breast cancer cells. Biochem. Biophys. Res. Commun.

[33] Shen D.W., Fojo A., Chin J.E., Roninson I.B., Richert N., Pastan I. (1986). Human multidrug-resistant cell lines: Increased mdr1 expression can precede gene amplification. Science.

[34] Bareford L.M., Swaan P.W. (2007). Endocytic mechanisms for targeted drug delivery. Adv. Drug Deliv. Rev.

[35] Zhang X., Chibli H., Kong D., Nadeau J. (2012). Comparative cytotoxicity of gold–doxorubicin and InP–doxorubicin conjugates. Nanotechnology.

[36] Wike-Hooley J.L., Haven J., Reinhold H.S. (1984). The relevance of tumour pH to the treatment of malignant disease. Radiother. Oncol.

[37] Raghunand N., Altbach M.I., van Sluis R., Baggett B., Taylor C.W., Bhujwalla Z.M., Gillies R.Z. (1999). Plasmalemmal pH gradients in drugsensitive and drug-resistant MCF-7 human breast carcinoma xenografts measured by 31P MR spectroscopy. Biochem. Pharmacol.

[38] Ojugo A.S., McSheehy P.M., McIntyre D.J., McCoy C., Stubbs M., Leach M.O., Judson I.R., Griffiths J.R. (1997). Measurement of the extracellular pH of solid tumours in mice by magnetic resonance spectroscopy: A comparison of exogenous 19F and 31P probes. NMR Biomed.

[39] Mark F., McCarty B.A., Julian W. (2010). Manipulating tumor acidification as a cancer treatment strategy. Alter. Med. Rev.

[40] Tannock I.F., Rotin D. (1989). Acid pH in tumors and its potential for therapeutic exploitation. Cancer Res.

[41] Sheetal D., Maheswara Reddy E., Asmita P., Varsha P., Anjali S., Prasad B.L.V. (2011). Cytotoxicity of sophorolipid-gellan gum-gold nanoparticle conjugates and their doxorubicin loaded derivatives towards human glioma and human glioma stem cell lines. Nanoscale.

[42] Rouhana L.L., Jaber J.A., Schlenoff J.B. (2007). Aggregation-resistant water-soluble gold nanoparticles. Langmuir.

[43] Narayanan K.B., Sakthivel N. (2008). Coriander leaf mediated biosynthesis of gold nanoparticles. Mater. Lett.

[44] Bankar A., Joshi B., Kumar A.R., Zinjarde S. (2010). Banana peel extract mediated synthesis of gold nanoparticles. Colloids Surf. B.

[45] Reddy A.S., Chen C.Y., Chen C.C., Jean J.S., Chen H.R., Tseng M.J., Fan C.W., Wang J.C. (2010). Biological synthesis of gold and silver nanoparticles mediated by the bacteria bacillus subtilis. J. Nanosci. Nanotechnol.

[46] Bhumkar D.R., Joshi H.M., Sastry M., Pokharkar V.B. (2007). Chitosan reduced gold nanoparticles as novel carriers for transmucosal delivery of insulin. Pharm. Res.

[47] Sheetal D., Maheswara Reddy E., Anjali S., Varsha P., Prasad B.L.V. (2008). Natural gum reduced/stabilized gold nanoparticles for drug delivery formulations. Chem. Eur. J.

[48] Xu Q., Mao C., Liu N.N., Zhu J.J., Sheng J. (2006). Direct electrochemistry of horseradish peroxidase based on biocompatible carboxymethyl chitosan–gold nanoparticle nanocomposite. Biosens. Bioelectron.

[49] Brust M., Walker M., Bethell D., Schiffrin D.J., Whyman R. (1994). Synthesis of thiol-derivatised gold nanoparticles in a two-phase liquid-liquid system. J. Chem. Soc. Chem. Commun.

[50] Hussain N., Jaitley V., Florence A.T. (2001). Recent advances in the understanding of uptake of microparticulates across the gastrointestinal lymphatics. Adv. Drug Deliv. Rev.

[51] Kohli A.K., Alpar H.O. (2004). Potential use of nanoparticles for transcutaneous vaccine delivery: Effect of particle size and charge. Int. J. Pharm.

[52] Yoo H.S., Lee K.H., Oh J.E., Park T.G. (2000). *In vitro* and *in vivo* anti-tumor activities of nanoparticles based on doxorubicin–PLGA conjugates. J. Control. Release.

[53] Liu Z., Sun X., Nakayama-Ratchford N., Dai H. (2007). Supramolecular chemistry on water-soluble carbon nanotubes for drug loading and delivery. ACS Nano.

[54] Liu Z., Fan A.C., Rakhra K., Sherlock S., Goodwin A., Chen X., Yang Q., Felsher D.W., Dai H. (2009). Supramolecular stacking of doxorubicin on carbon nanotubes for *in vivo* cancer therapy. Angew. Chem. Int. Ed.

[55] Aryal S., Grailer J.J., Pilla S., Steeber D.A., Gong S.Q. (2009). Doxorubicin conjugated gold nanoparticles as water-soluble and pH-responsive anticancer drug nanocarriers. J. Mater. Chem.

[56] Sunil P., Goldie O., Ashmi M., Ritu S., Mukeshch T., Madhuri S. (2013). Folic acid mediated synaphic delivery of doxorubicin using biogenic gold nanoparticles anchored to biological linkers. J. Mater. Chem. B.

[57] Prabaharan M., Grailer J.J., Pilla S., Steeber D.A., Gong S.Q. (2009). Gold nanoparticles with a monolayer of doxorubicin-conjugated amphiphilic block copolymer for tumor-targeted drug delivery. Biomaterials.

[58] Bhujwalla Z.M., Artetmov D., Bllestros P., Cerdan S., Gillies R.J., Solaiyappan M. (2002). Combined vascular and extracellular pH imaging of solid tumors. NMR Biomed.

[59] Swietach P., Hulikova A., Patiar S., Vaughan-Jones R.D., Harris A.L. (2012). Importance of intracellular pH in determining the uptake and efficacy of the weakly basic chemotherapeutic drug, doxorubicin. PLoS One.

[60] Maeda H. (2001). The enhanced permeability and retention (EPR) effect in tumor vasculature: The key role of tumor-selective macromolecular drug targeting. Adv. Enzym. Regul.

[61] Hobbs S.K., Monsky W.L., Yuan F., Roberts W.G., Griffith L., Torchilin V.P., Jain R.K. (1998). Regulation of transport pathways in tumor vessels: Role of tumor type and microenvironment. Proc. Natl. Acad. Sci. USA.

[62] Patel N.K., Sinha V.K. (2009). Characterization and optimization of water-soluble chitosan derivatives. Int. J. Polym. Mater.

