# Supplementary material for: Efficient pH Dependent Drug Delivery to Target Cancer Cells by Gold Nanoparticles Capped with Carboxymethyl Chitosan
Source: Int J Mol Sci. 2014 May 9;15(5):8216–34. doi: 10.3390/ijms15058216 (PMC4057728; doi:10.3390/ijms15058216)

Supplementary Information

Figure S1. UV-visible absorption spectra of AuNPs stabilized in CM-Chitosan by (**A**) Various concentrations of CM-Chitosan; (**B**) Various concentrations of HAuCl4; and (**C**) Varying the time from 2 to 10 min.

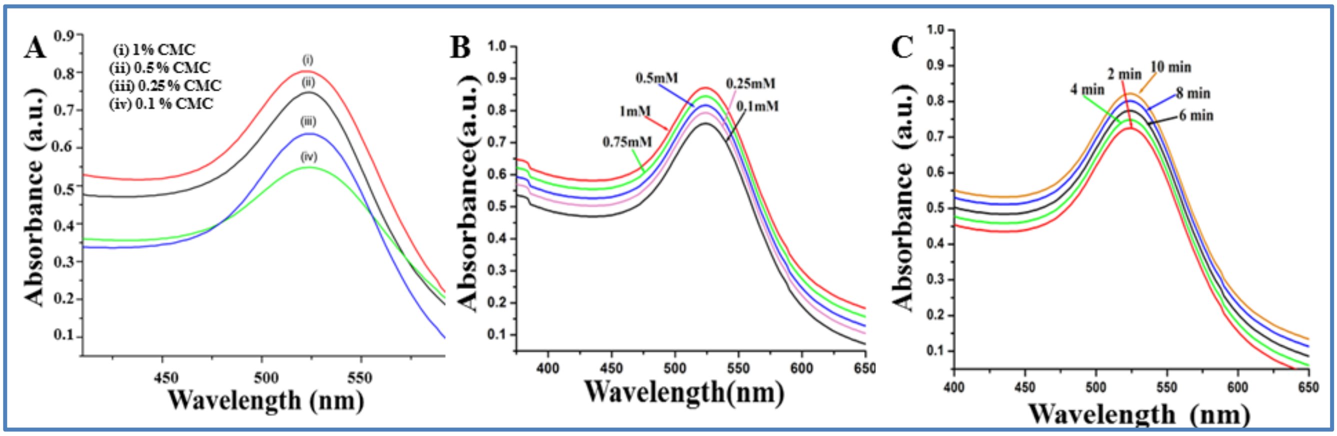

Figure S2. Zeta potential of CM-Chitosan capped AuNPs (**A**) and DOX loaded AuNPs (**B**).

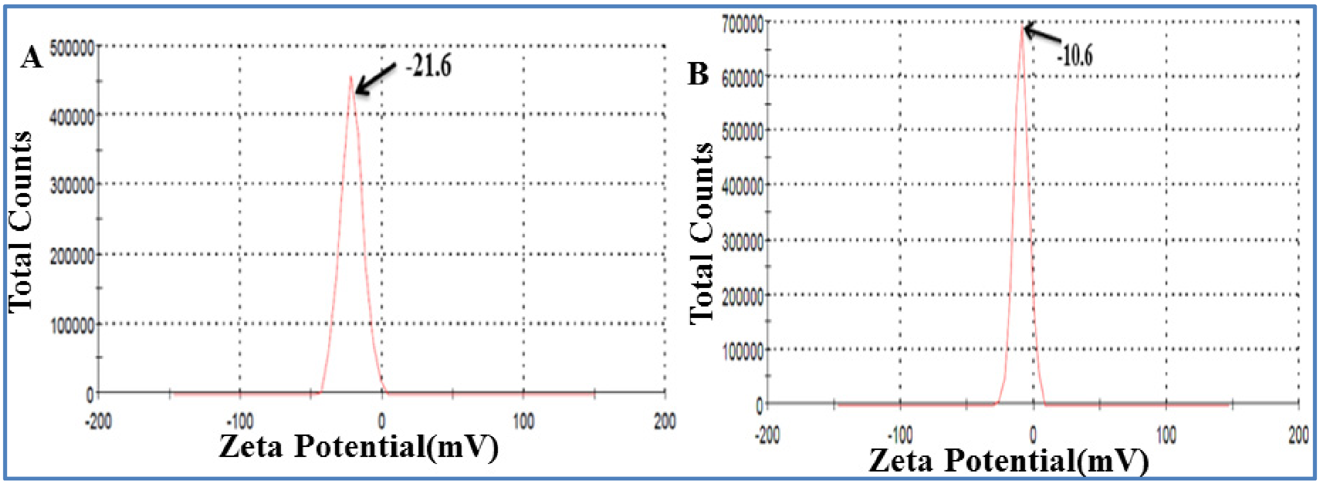

Figure S3. Size distribution cure of AuNPs (**A**) and DOX loaded AuNPs (**B**) from dynamic light scattering.

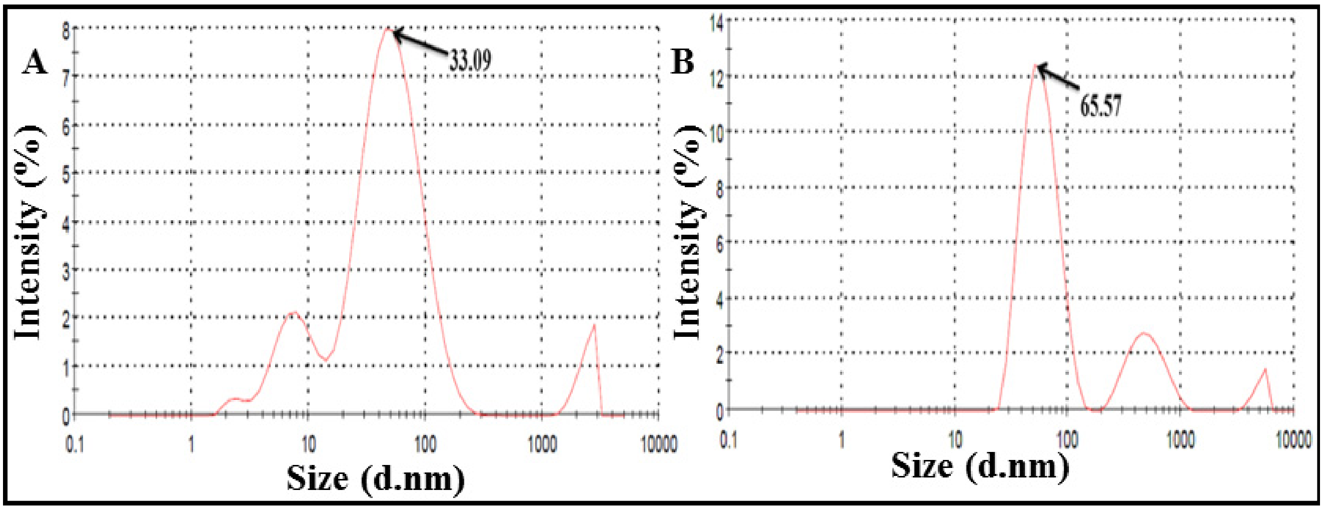

Supplement: Supplementary file 1 [file ijms-15-08216-s001.pdf]
